# Letter to editor regarding “The morphology of the proximal femur in cementless short-stem total hip arthroplasty: no negative effect on offset reconstruction, leg length difference and implant positioning”

**DOI:** 10.1186/s13018-022-03119-z

**Published:** 2022-04-07

**Authors:** Jimin Ma, Jiale Li, Le Cao, Kai Sun, Haitao Yang, Haitao Fan

**Affiliations:** Department of Orthopedics, Fuyang Hospital of Anhui Medical University, Fuyang, Anhui China

Dear Editor,

Recently, we read with great interest the article “The morphology of the proximal femur in cementless short-stem total hip arthroplasty: No negative effect on offset reconstruction, leg length difference and implant positioning” by Luger et al. [1]. We much appreciate the authors’ work in this field; however, we have some of our concerns regarding the article and would like to discuss them with the authors.

Firstly, Dorr and Noble classification are the most classical classification standards for anatomical shape of the proximal femur [2, 3]. The authors reported that the anatomical shape of the proximal femur was determined according to the Dorr classification by two reviewers. As we all know, the classical Dorr classification is judged by the visual judgment according to the reviewers. However, several reports [4–6] have shown that the proximal femoral shape can be grouped according to the femoral cortical index (FCI): > 0.6 were Dorr type A, ≤ 0.6 and ≥ 0.5 were Dorr type B, and < 0.5 were Dorr type C. Why not use a more objective and quantifiable method than visual judgment? Noble classification [3] is also a good choice.

Secondly, leg length difference (LLD) after total hip arthroplasty (THA) does not only mean lengthening of operative limb, but also the shortening of operative limb. Lim et al. [4] conducted statistical analysis was made not only for LLD > 5 mm or > 10 mm, but also for LLD < -5 mm or < -10 mm. However, in the authors' report, logistic regression for LLD ≥ 5 mm or ≥ 10 mm showed no difference in Dorr type and concluded that proximal femur morphology had no negative effect on LLD. We don't think this conclusion is appropriate. Whether the LLD in the author's article stands for lengthening, shortening, or absolute value, it would be better to discuss it separately.

Finally, the postoperative LLD was least obvious in Dorr A and most obvious in Dorr C. This is the opposite of what previous studies have shown [4, 7, 8]. However, we found no explanation or discussion of this phenomenon in the discussion section of this article.

## Data Availability

All data generated or analyzed during this study are included in this published article.

## Abbreviations

FCI: Femoral cortical index
LLD: Leg length difference
THA: Total hip arthroplasty
